# Double Duty: Complete Pathologic Response of Two Colonic Primaries with Mosaicism of a Novel *MLH1* Mutation to Neoadjuvant Pembrolizumab

**DOI:** 10.3390/curroncol30100653

**Published:** 2023-10-06

**Authors:** Beatrice Preti, Laila Schenkel, Matthew Cecchini, Tommaso Romagnoli, Michael Susmoy Sanatani, Karissa French, Patrick Colquhoun, Mark David Vincent

**Affiliations:** 1 Division of Medical Oncology, Department of Oncology, Western University, London, ON N6A 3K7, Canada; michael.sanatani@lhsc.on.ca (M.S.S.); mark.vincent@lhsc.on.ca (M.D.V.); 2 Division of Molecular Diagnostics, Western University, London, ON N6A 3K7, Canada; laila.schenkel@lhsc.on.ca; 3 Department of Pathology and Laboratory Medicine, Western University, London, ON N6A 3K7, Canada; matthew.cecchini@lhsc.on.ca (M.C.); tommaso.romagnoli@lhsc.on.ca (T.R.); 4 Schulich School of Medicine & Dentistry, Western University, London, ON N6A 3K7, Canada; 5 Temerty Faculty of Medicine, University of Toronto, Toronto, ON M5S 1A1, Canada; 6 Department of Surgery, Western University, London, ON N6A 3K7, Canada; patrick.colquhoun@lhsc.on.ca

**Keywords:** colon cancer, MLH1, immunotherapy, dMMR, MSI-H

## Abstract

We present a fascinating case of a 57-year-old male with a novel mutation in MLH1 (MLH1:c.1288G > T, p.(Glu430*)), who presented with two synchronous colonic tumours, initially deemed unresectable, and experienced a complete pathological response on neoadjuvant pembrolizumab. Extensive genetic testing revealed post-zygotic mosaicism from the novel mutation.

## 1. Introduction

The benefit of immunotherapy in metastatic colorectal cancer with high microsatellite instability/deficient mismatch repair (MSI-H/dMMR) has been established to enhance progression-free survival and response rates compared to first-line chemotherapy. However, the role of immunotherapy in the neoadjuvant rectal or colon cancer setting is less well understood. Smaller studies have suggested a role for immunotherapy in this setting, but details on the long-term outcomes and benefits remain elusive. Even for MSI-H rectal cancers, the standard of care remains surgical resection, usually with neoadjuvant chemoradiotherapy or chemotherapy if the tumour is locally advanced. There is also no standard approach for borderline resectable colon cancers, and systemic chemotherapy is often used to “downstage” a cancer to make it resectable. There is, therefore, a need to define the role that immunotherapy can or should play in the management of MSI-H, locally advanced, or borderline resectable colon and rectal cancers where a good tumour response may lead to curative (rather than palliative) intent treatment.

In this report, we present the case of a previously healthy 57-year-old male with post-zygotic mosaicism of an *MLH1* mutation who developed two synchronous, initially unresectable, MSI-H/dMMR colonic tumours. He experienced cessation of bleeding after one cycle of pembrolizumab, exhibited lowered tumour markers after two cycles, and the tumour had a complete pathological response after six cycles. In this report, we also describe a rare mutation in *MLH1*, MLH1:c.1288G > T, p.(Glu430*), a nonsense mutation predicted to create a stop codon in exon 12 and result in a loss of the MLH1 function, which was one of the two *MLH1* mutations found in this patient.

## 2. Detailed Case Description

A 57-year-old male of Korean origin with no relevant past medical history presented to general surgery in February 2021 with a one-and-a-half-year history of abdominal discomfort in the left lower quadrant. A workup by the family physician had revealed a microcytic anaemia. CT (computed tomography) scans showed two masses, one in the sigmoid colon measuring 7.4 cm × 6.5 cm and one in the ascending colon measuring 3.5 cm × 2.2 cm, as well as an adjacent soft tissue mass in the right abdomen measuring 3.0 × 4.0 × 2.4 cm, thought to likely represent an involved lymph node. Additionally, a 0.8 × 0.7 cm mass in the liver was reported, favoured to represent a cyst, as well as another mass in the left buttock, favoured to represent an epidermal inclusion cyst. The clinical impression was that of two synchronous primaries, without metastatic disease. 

The patient underwent colonoscopy, which revealed a nearly obstructing sigmoid mass at the 25 cm mark. The scope was unable to be inserted past the obstructing mass; thus, the proximal ascending colon mass was unable to be reached or biopsied. A biopsy of the sigmoid lesion showed a well-differentiated adenocarcinoma with a loss of nuclear expression in *MLH1* and *PMS2*, without a BRAF V600E mutation. This identified the tumour as one with high microsatellite instability/deficient mismatch repair (hereafter termed MSI-H/dMMR).

The patient then underwent a diverting loop ileostomy in March 2021, shortly after diagnosis, due to impending obstruction. This procedure was the consequence of a diagnostic laparoscopy that demonstrated growth into the anterior abdominal wall, thus marking both tumours as unresectable. Treatment by medical oncology was planned for pembrolizumab; however, the patient developed significant rectal bleeding before treatment could commence. His performance status dropped to borderline levels for systemic therapy, and he required a blood transfusion. He was admitted to the hospital for one week, during which time he received his first dose of pembrolizumab (planned at this point as a palliative-intent therapy due to the unresectable nature of the tumours) as an inpatient. He also required a left nephrostomy tube due to decreased left-sided renal perfusion and left renal hydronephrosis, a procedure that was complicated by perforation of the renal pelvis. The patient’s rectal bleeding had resolved by the time of discharge, and he had maintained stable haemoglobin levels for several days after only one dose of pembrolizumab and without any local or other therapies.

After two cycles of pembrolizumab, the patient’s CA-19, found to be 41 pre-treatment and 25 in April 2021, had become undetectable. CEA remained low. He experienced no further bleeding in the rectum. The main adverse effect of treatment he experienced was pruritis, which was treated with hydroxyzine and topical hydrocortisone.

**Figure 1:**
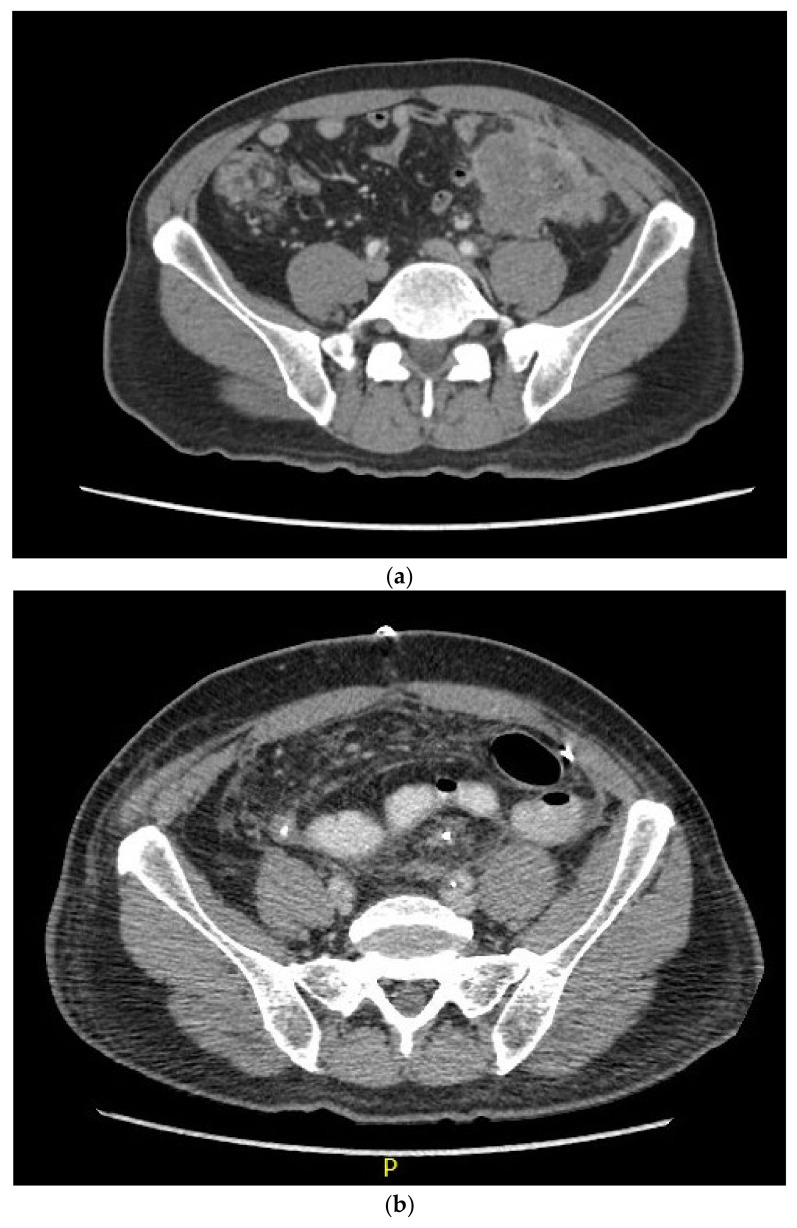
CT scans from (**a**) February 2021, pre-treatment, and (**b**) October 2021, pre-surgery.

After six cycles of pembrolizumab, the patient’s tumours were no longer palpable on physical examination. Repeat CT scans in August 2021 (Figure 1) showed an interval decrease in the size of both tumours as well as the lymph nodes, potentially a complete response, although a commitment was not made at the time. At this point, the decision was made to attempt surgical resection, which allowed the patient to be taken to the operating theatre for total abdominal colectomy, en bloc resection of the left abdominal wall and left ureter, ileorectal anastomosis, cystoscopy with retrograde pyelogram and bilateral ureteric stent placement, and reconstruction of the left ureter with a Boari primary flap in October 2021. The patient tolerated this procedure well, without significant post-operative complications.

**Figure 2:**
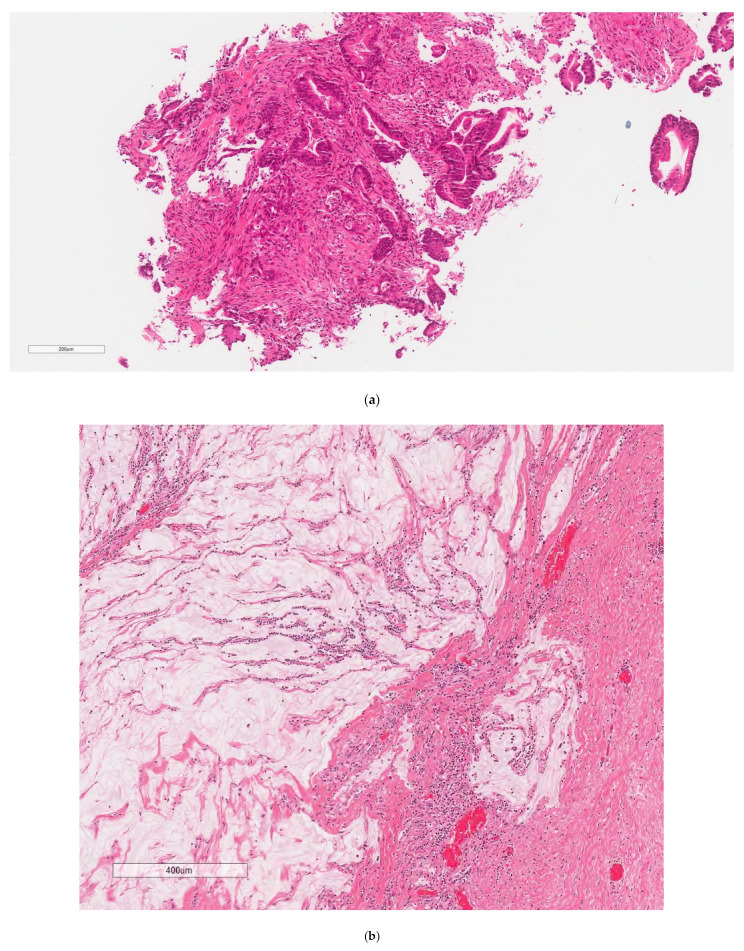

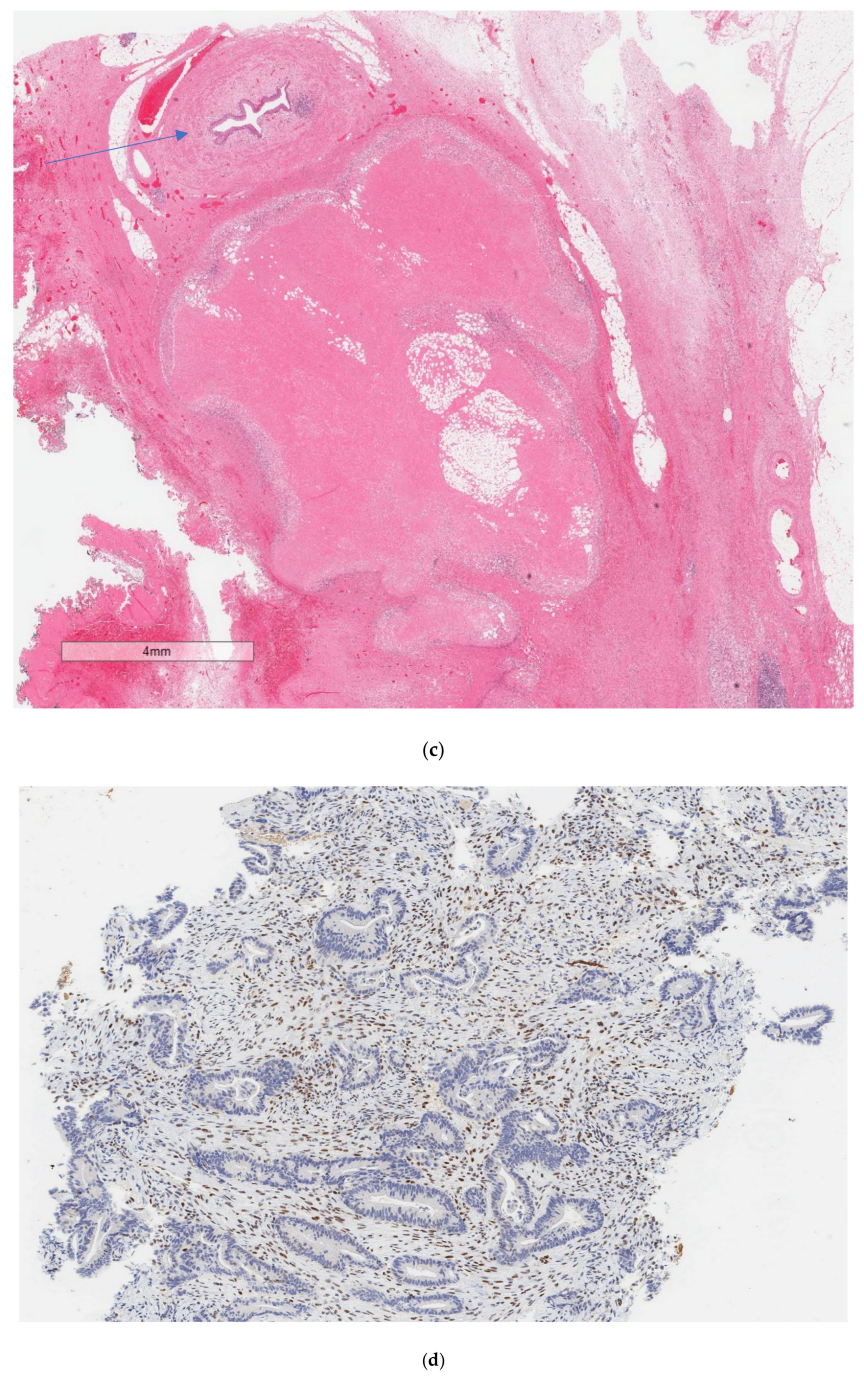

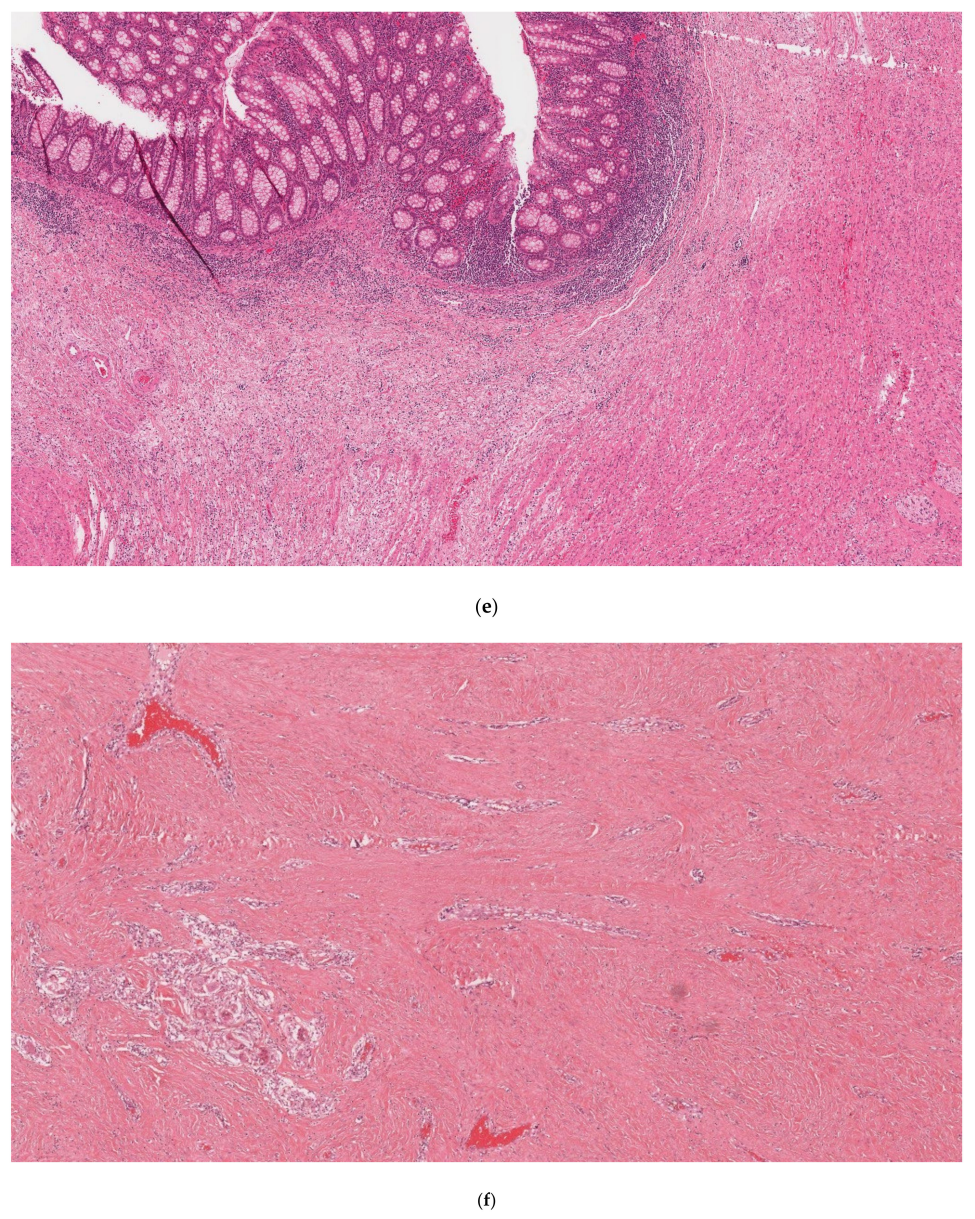
Pathology from tumour sites, as listed, (**a**) Sigmoid colon, biopsy. Invasive, well-differentiated adenocarcinoma. H&E, (**b**) Irregular mucin pools with a fibroinflammatory reaction, and no residual neoplastic cells were identified. H&E, (**c**) Left ureter (arrow) and mass. This mass likely represents a previously positive lymph node that has been entirely replaced with foamy histiocytes and fibrosis. No residual tumour was identified. H&E, (**d**) MLH1 = loss of nuclear expression. There is intact nuclear expression within background nonneoplastic tissue, (**e**) Mass 1 = proximal ascending colon, which is likely the site of the previous tumor. There is an area of submucosal and intramuscular fibrosis with associated lymphohistiocytic inflammation. The area is surrounded by large, reactive lymphoid aggregates (not pictured). There is no evidence of dysplasia or residual tumor, (**f**) Mass 2 = sigmoid colon, which is likely the site of the previous tumor. There is an area of fibrosis with chronic xanthogranulomatous inflammation. There are associated occasional acellular mucin pools (not pictured). No residual viable tumor or evidence of dysplasia is present.

Pathology from this surgery (Figure 2) confirmed two primary tumour locations; however, surprisingly, there was no residual/viable tumour in either location, thus indicating a complete pathologic response of both tumours to neoadjuvant pembrolizumab. Pathology from the ascending colon mass demonstrated an area of submucosal and intramuscular fibrosis, associated with scattered lymphohistiocytic inflammation, surrounded by several large reactive lymphoid aggregates. A necrotic lymph node was visualized in the pericolic fat, and the 26 lymph nodes identified were all negative for malignancy. Pathology from the sigmoid colon mass demonstrated fibrosis, chronic xanthogranulomatous inflammation, and occasional areas with empty mucin pools. This was suggestive of a previous tumour site with no residual/viable tumour cells. The thirteen lymph nodes identified were all negative for malignancy. This represents a complete pathological response from the neoadjuvant pembrolizumab.

An uninvolved bowel was noted to contain prominent mucosal reactive lymphoid aggregates.

The patient continued to do well for 20 months from the time of surgery, with no evidence of disease recurrence. He continued pembrolizumab in the adjuvant setting for a total of two years of treatment and now continues surveillance.

Just prior to the discontinuation of pembrolizumab, the patient presented to a clinic with chest pain. The initial concern was for a pembrolizumab-induced cardiac complication, such as myocarditis and pericarditis, and an urgent cardiology opinion was solicited. However, before the outpatient care and a full workshop could be coordinated, the patient presented to the emergency department with worsening chest pain and was found to have an STEMI with 99% occlusion of the LAD. He underwent angioplasty and stenting and made a full recovery. The episode was not felt to be related to his malignancy. 

## 3. Genetic Case Presentation

Next-generation sequencing was performed on the biopsied tumour in February 2021. This demonstrated two mutations in MLH1. The first was a nonsense mutation *1288G > T*, *p*.(Glu430*), which is predicted to create a stop codon in exon 12 and result in premature protein truncation. This mutation has not previously been reported in the literature or listed in any database, to our knowledge. Additionally, there was a *1852_1854delAAG*, *p*.(Lys618del) mutation reported, which is an in-frame deletion variant previously reported in families affected by Lynch syndrome/Hereditary Nonpolyposis Colorectal Cancer (HNPCC). Additionally, *MLH1* promotor methylation was not detected in the sample. This combination of the MSI-H/dMMR status, presence of synchronous dual primaries (although the more proximal tumour did not have tissue confirmation of MSI status), lack of detected *MLH1* promoter methylation, two synchronous *MLH1* mutations, and lack of BRAF mutation together led to a referral to genetics to formalise the diagnosis of Lynch syndrome/HNPCC in the patient. 

In December 2021, the patient underwent his first formal genetic testing with routine genetic testing as well as consultation with a genetics counsellor. Next-generation sequencing performed on germline DNA via a blood sample did not detect a pathogenic variant in the five tested genes on the standard Lynch syndrome/HNPCC panel. Somatic next-generation sequencing performed on the tumour tissue showed two biallelic MLH1 variants, a nonsense mutation and an in-frame deletion, specific to the patient’s tumour DNA, favouring a sporadic cause of the patient’s malignancy. At this point, it was felt that genetic results were not suggestive of a diagnosis of Lynch Syndrome/HNPCC, as the mutations from the tumour were not identified in the blood on the standard panel.

However, given the fact that at least one of the mutations had not previously been reported in the literature, we repeated the next-generation sequencing in the blood, this time looking specifically for the *MLH1:c.1288G > T*, *p*.(Glu430*) mutation, and found this to be present at a rate of 2.85%. This is below our centre’s normal detection level of 5% and was suggestive of post-zygotic (somatic) mosaicism. To further confirm this diagnosis, sequencing was performed on a sample of normal, non-malignant colons, revealing the *MLH1:c.1288G > T*, *p*.(Glu430*) mutation at a rate of 10.3%. The second mutation, *1852_1854delAAG*, *p*.(Lys618del), was not identified during any of this testing.

Following this diagnosis, the patient’s two daughters underwent genetic testing to look for the rare mutation. This was not identified in either of the patient’s daughters, thus suggesting the mutation was not transmitted/inherited from the patient to his offspring.

## 4. Discussion

This case presents, to our knowledge, the first example of pembrolizumab being used de facto as neoadjuvant therapy in two synchronous colon cancer primaries initially thought to be unresectable to obtain a complete pathological response at the time of surgery.

The current role of immunotherapy in colon cancer has been established in the metastatic domain. Two recent landmark studies have solidified the role for immunotherapy in MSI-H/dMMR tumours. The KEYNOTE-177 trial demonstrated the benefit of first-line pembrolizumab over chemotherapy in metastatic MSI-H/dMMR cancers [1] and is now, in fact, routinely used in many centres. The phase II CHECKMATE-142 trial likewise demonstrated the benefit of first-line ipilimumab plus nivolumab in metastatic MSI-H/dMMR cancers [2]. Finally, the phase II Canadian Cancer Trials Group CO.26 trial demonstrated a 2.5-month survival benefit for durvalumab and tremelimumab in patients with advanced, treatment-refractory colon cancer when compared to the best supportive care alone [3].

In the neoadjuvant setting, the role of immunotherapy is less well defined. A recently reported study treated 40 patients with early-stage colon cancer, half with MSI-high tumours, with one dose of ipilimumab and two doses of nivolumab in the neoadjuvant setting [4]. Of the twenty patients with MSI-high tumours, nineteen had major pathological responses with 10% or less of viable tumour tissue remaining, and twelve had complete pathological responses [4]. However, none of these patients are documented as having two synchronous colonic primaries.

A phase II trial evaluated the role of perioperative pembrolizumab in non-metastatic MSI-H/dMMR non-metastatic tumours, including colon cancer [5]. Among 30 patients with evaluable disease, 9 experienced a complete response, although the final results from this study are pending.

A recent Chinese case series demonstrated a neoadjuvant immunotherapy in two patients with colon cancer; however, the pathological response could not be determined due to the lack of measurable disease [6]. Most recently, a phase II trial demonstrated complete clinical response in 12 patients with localised MSI-H/dMMR rectal tumours [7]. However, all of these neoadjuvant studies appear to have been conducted in patients with only one primary tumour at the time of presentation.

Additionally, we present in this case report an example of post-zygotic mosaicism from a rare mutation in MLH1, MLH1:c.1288G > T, p.(Glu430*), a nonsense mutation predicted to create a stop codon in exon 12. However, we continue to attempt to understand the significance of the two *MLH1* mutations in this patient’s case, as, due to the nearly obstructing nature at presentation and the pathological complete response, we do not have pathology from the second, more proximal tumour. It is assumed that this is a case of dual primaries, although a skip metastasis (though unusual from left to right) cannot be entirely excluded; consequently, the former is what we have assumed in this report, although the patient did receive a full two years of pembrolizumab prior to beginning surveillance only. It is likely that the post-zygotic mosaicism of MLH1 might have predisposed the patient to develop two synchronous tumours.

Few cases of post-zygotic mosaicism of MLH1 exist in the literature. The available information includes a case of an MLH1 (c.518_519del; *p*.(Tyr173Trpfs*18)) mutation in a 31-year-old man with no family history or genetic history, with a frequency of 13% in the blood, leading to rectosigmoid adenocarcinoma [8]. A case of a double somatic mutation, one with mosaicism (the case with our patient), has also been documented in the literature [9]. Based on this case, which described mosaicism transmission to the patient’s offspring, we pursued testing of our patient’s offspring, which, as mentioned above, was negative.

An additional facet of this case that is worth noting is the initially negative genetic testing, which is the result of a fixed cutoff of 5% of a mutation in the blood sample, which is standard at our centre. While this practice may reduce false positives, as in any test, an adjustment of the values deemed “positive” will have an effect on the false negative rate as well. Especially in the case of mosaicism, the levels of mutations found in the bloodstream may be well below this cutoff and still be clinically significant if a mutation leads to local oncogenesis or affects the reproductive organs.

In summary, our case highlights several thought-provoking and hypothesis-generating circumstances:

1. The higher response rate with immunotherapy compared to chemotherapy in MSI-H colorectal cancers may suggest that ICI treatment should be considered as first line in the case of borderline resectable tumours, and randomized clinical trials comparing the current standard of chemo (radio-)therapy to immunotherapy for MSI-H tumours should be initiated.
2. Multiple primary tumours may be present in MSI-H mosaicism and be associated with varying mutations, and yet they may all respond to ICI therapy.
3. Cognitive biases such as confirmation bias may team up with a testing strategy set up to avoid excessive false positive rates and lead to missed diagnoses of a rare constellation of findings. Revisiting and identifying assumptions made during the diagnostic workup and identifying the link in the testing chain where a possible true positive result may have been hidden by a test cutoff are indicated whenever the clinical course does not match the suggested initial test result.

## 5. Conclusions

We present a fascinating case of a post-zygotic mosaicism of MLH1 in a 57-year-old male with dual, synchronous mutations in MLH1 (including *MLH1:c.1288G > T*, *p*.(Glu430*)). The patient presented with dual, synchronous colonic tumours, initially deemed unresectable due to abdominal wall invasion. He experienced a substantial tumour response on pembrolizumab, which allowed for the consideration of surgery. He pathology demonstrated a complete pathological response at both tumour sites from neoadjuvant pembrolizumab. Extensive genetic testing revealed a likely post-zygotic mosaicism event associated with a Lynch/HNPCC-like cancer phenotype in this patient. Further education around this phenomenon will help raise awareness around the possibility of mosaicism, its possible transmission to offspring, and its role in oncogenesis, as well as around the limits of genetic testing based on blood sampling. Future studies should formally test the neoadjuvant use of ICI therapy prospectively in locally advanced colon cancer.

## Data Availability

No new data were generated by this report.
